# Application of a disease-specific mapping function to estimate utility gains with effective treatment of schizophrenia

**DOI:** 10.1186/1477-7525-3-57

**Published:** 2005-09-11

**Authors:** Leslie A Lenert, Marcia FT Rupnow, Christine Elnitsky

**Affiliations:** 1 Veterans Administration San Diego Health Care System, San Diego, California, USA; 2 University of California, San Diego, California, USA; 3 Janssen Medical Affairs, L.L.C., Titusville, NJ, USA; 4 Health Services Research and Development Service, Department of Veteran Affairs, Washington, DC, USA

## Abstract

**Background:**

Most tools for estimating utilities use clinical trial data from general health status models, such as the 36-Item Short-Form Health Survey (SF-36). A disease-specific model may be more appropriate. The objective of this study was to apply a disease-specific utility mapping function for schizophrenia to data from a large, 1-year, open-label study of long-acting risperidone and to compare its performance with an SF-36-based utility mapping function.

**Methods:**

Patients with schizophrenia or schizoaffective disorder by DSM-IV criteria received 25, 50, or 75 mg long-acting risperidone every 2 weeks for 12 months. The Positive and Negative Syndrome Scale (PANSS) and SF-36 were used to assess efficacy and health-related quality of life. Movement disorder severity was measured using the Extrapyramidal Symptom Rating Scale (ESRS); data concerning other common adverse effects (orthostatic hypotension, weight gain) were collected. Transforms were applied to estimate utilities.

**Results:**

A total of 474 patients completed the study. Long-acting risperidone treatment was associated with a utility gain of 0.051 using the disease-specific function. The estimated gain using an SF-36-based mapping function was smaller: 0.0285. Estimates of gains were only weakly correlated (r = 0.2). Because of differences in scaling and variance, the requisite sample size for a randomized trial to confirm observed effects is much smaller for the disease-specific mapping function (156 versus 672 total subjects).

**Conclusion:**

Application of a disease-specific mapping function was feasible. Differences in scaling and precision suggest the clinically based mapping function has greater power than the SF-36-based measure to detect differences in utility.

## Background

Estimation of cost-effectiveness in clinical trial settings requires measurement of changes in utility. This is particularly difficult in diseases that impact cognitive functioning, such as schizophrenia or Alzheimer's disease, because these impairments may preclude direct elicitation of utilities in trial participants. Even in cognitively intact persons, direct elicitation often is logistically difficult in clinical trial settings and therefore rarely done. To overcome these issues, researchers have developed health index models to assign a utility to each individual. Health index models, including the Health Utilities Index, EuroQol (EQ-5D), and the Quality of Well-Being Scale (QWB), present comprehensive models of quality of life [1-4]. Measurement of attributes within these models allows assignment of utility scores based on population models of values summarized by the index; however, if these measures were not performed during the study, the direct application of a health index model is not possible.

Many trials include measurements of health status performed with short-form measures such as the 36-Item Short-Form Health Survey (SF-36) [5,6]. An alternative to health index models is to use these data to estimate utility values. Efforts in this vein began with work by Fryback and colleagues on mapping between the SF-36 and the QWB [7] and have been extended by many others [8,9]. A second approach, described by Brazier and colleagues, has been to develop a health index based on the content of a short-form measure and to measure utilities for the models with larger numbers of states as defined by the short-form measure [10,11]. A refinement of this method, which involves using k-means clustering to find a small number of states that represent patterns of disease effects on health status, has been described by Lenert and colleagues [12]. The primary advantage of this approach is that it allows direct comparison of implications of differences between patient and general population values, as is recommended by the cost-effectiveness analysis guideline panel [13]. In comparison studies, this approach appeared to be more responsive than other mapping functions for short-form measures in depression [14].

Use of a health index model or a short-form measure of health status does not address the issue of disease-specific effects on quality of life, however. To capture disease-specific effects, investigators typically measure disease activity with validated scales specific to a disorder. Other times, modeling of the symptoms of the disease itself may be required. In schizophrenia, a commonly used disease activity measurement scale is the Positive and Negative Syndrome Scale (PANSS) [15]. This scale measures, via interview, the total burden of symptoms and impact of disease. Change in the PANSS may or may not be reflected in short-form measures such as the SF-36, although they often are responsive in schizophrenia and other mental illnesses. In this paper, we describe the application of a disease-specific utility mapping function based on the PANSS, incorporating an additional assessment of the impact of adverse effects of medication into the model. The methods used to create the model have been described elsewhere [16,17]. Briefly, a set of disease states for schizophrenia were developed from the PANSS based on data from a large, 1-year study that prospectively compared oral risperidone with conventional antipsychotic agents among patients with schizophrenia who were treated under usual practice conditions [18]. Data were analyzed by cluster analysis for five factor domains; cluster analysis results were compared with an expert-developed conceptual framework of disease states. Using a combination of the empirical data and the conceptual framework, eight disease states with varying levels of positive, negative, and cognitive impairment were established. Health states were described in a holistic fashion that included interactions between effects of symptoms of the disease and other aspects of quality of life. Utilities were measured in the general public from a convenience sample of a large Internet survey panel [19]. Participants viewed digital videos of actors depicting the eight health states and five common antipsychotic side effects (akathisia, pseudo-parkinsonism, orthostatic hypotension, dyskinesia, and clinically important weight gain) and rated the states using a visual analog scale (VAS) followed by a standard gamble (SG). The mean utility rating for each state and the reduction in utility with each adverse effect were estimated by re-weighting responses so that calculated mean values matched US population demographic profiles in age, ethnicity, and gender [17].

We report application of this mapping function to a 50-week, multicenter, international, open-label study of long-acting risperidone in patients with schizophrenia and schizoaffective disorder. Detailed safety and efficacy results of this study have been published and presented elsewhere [20,21]. Changes in utility associated with long-term use of long-acting risperidone were estimated using the mapping function in patients who completed 50 weeks of therapy. We then compared results to a mapping function for the SF-36.

## Methods

To assess the responsiveness of the utility mapping function, we applied it to data from an open-label, international (Europe and Canada), 50-week trial evaluating the long-term safety and tolerability of long-acting risperidone in 725 patients with schizophrenia or schizoaffective disorder considered to be stable at study entry [20,21]. The final protocol for, and any amendments to, the original study were reviewed and approved by independent Ethics Committees or by appropriately constituted institutional review boards (IRBs) according to specifications outlined in the US Code of Federal Regulations (CFR). This trial was conducted in accordance with current International Conference on Harmonisation (ICH)-Good Clinical Practice guidelines and the Declaration of Helsinki and its subsequent revisions. Patients were aged 18 to 85 years with a diagnosis of schizophrenia or schizoaffective disorder according to DSM-IV criteria [22] and were judged to be clinically stable (stable symptoms and antipsychotic dose for at least 1 month).

## Trial medication

During a 2-week run-in period, antipsychotics other than risperidone were discontinued, and patients not currently being treated with risperidone received flexible doses of 1 to 6 mg/daily of oral risperidone. Assessments performed prior to this run-in period, however, were considered as the baseline for this analysis. By protocol, pharmacokinetic considerations, and investigator judgment, patients were assigned to flexible-dose treatment with 25, 50, or 75 mg long-acting risperidone given by intramuscular gluteal injection every 2 weeks. The investigator could adjust the dose of long-acting risperidone when necessary. Medications other than long-acting risperidone that could be initiated or continued during the trial included anticholinergic agents, antidepressants, mood stabilizers, propranolol for akathisia, and benzodiazepines for agitation and insomnia.

## Assessments

PANSS total scores [15] and health-related quality-of-life assessments, measured by the SF-36 [5,6], were collected at weeks 1, 12, 24, 36, 50, and at endpoint. Adverse effects of treatment were assessed at the same time points, using the Extrapyramidal Symptom Rating Scale (ESRS) [23] to determine the Clinical Global Impression (CGI) of severity of parkinsonism, dystonia, and dyskinesia, and adverse-effect reporting to document weight gain and hypotension. Values for systolic blood pressure, pulse, and weight were obtained at baseline and endpoint.

## Analysis plan

Disease states were assigned at each observation based on the mean value of each patient's PANSS items using the model developed by Mohr and colleagues [16]. For patients completing at least 50 weeks of treatment, the mean values of VAS and SG utility were calculated from the PANSS, and VAS and SG utility were calculated from the SF-36. VAS and SG utilities calculated from the PANSS were adjusted for adverse effects using a multiplicative model. An individual with a score of ≥4 on any of the ESRS subscales for parkinsonism, dystonia, or dyskinesia was ascribed as having that adverse effect. An individual was ascribed as having orthostatic hypotension for the entire duration of the study if upon exit from the trial, the patient exhibited a ≥20-mm Hg drop in standing blood pressure. Patients with a gain of ≥10 kg (≥22.4 lb) during the study were assessed a utility tariff for weight gain. These measurements were performed only at 24 weeks, 50 weeks, and endpoint. Missing data were estimated using a last-value-carried-forward approach in patients completing the study.

Estimated utility values at each point in time were compared using the Wilcoxon signed rank test. Overall gains in utility over the course of the study were calculated by subtracting baseline from endpoint values and compared with those estimated from an SF-36-based mapping function developed by Nichol and colleagues [8]. These calculations were used to compare both the magnitude of the estimated utility gains and the correlation of the gains between the two mapping functions. To compare the responsiveness of the measures, we took the standard deviation of SF-36-based and clinically based utility measures at baseline and endpoint and the effect size seen in this study for each measure and estimated the sample size required for a clinical trial to confirm the effect seen in this observational study.

## Results

A total of 725 symptomatically stable patients with schizophrenia (n = 615) or schizoaffective disorder (n = 110), received long-acting risperidone treatment. Four hundred seventy-four patients (65.3%) completed the trial. Demographic characteristics of patients who completed or discontinued the study are displayed in Table 1. The only significant difference in baseline characteristics between those who completed the study and those who discontinued was the mean age.

**Table 1:** Demographics and Baseline Disease Characteristics for Patients Who Completed or Discontinued the Study

**Parameter**	**Patients Who Completed the Study (n = 474)**	**Patients Who Discontinued (n = 251)**	*P *value Between Groups*
Age, y (mean ± SE)	43.7 ± 0.7	39.3 ± 0.9	<0.0001
Sex			0.730
Female, n (%)	162 (34.2)	89 (35.5)	
Male, n (%)	312 (65.8)	162 (64.5)	
Race			0.502
Caucasian, n (%)	440 (92.8)	231 (92.0)	
Black, n (%)	8 (1.7)	8 (3.2)	
Asian, n (%)	6 (1.3)	5 (2.0)	
Hispanic, n (%)	4 (0.8)	2 (0.8)	
Other, n (%)	16 (3.4)	5 (2.0)	
Diagnosis			0.650
Schizophrenia, n (%)	400 (84.4)	215 (85.7)	
Schizoaffective disorder, n (%)	74 (15.6)	36 (14.3)	

SE indicates standard error. *Chi-square test.

**Figure 1:**
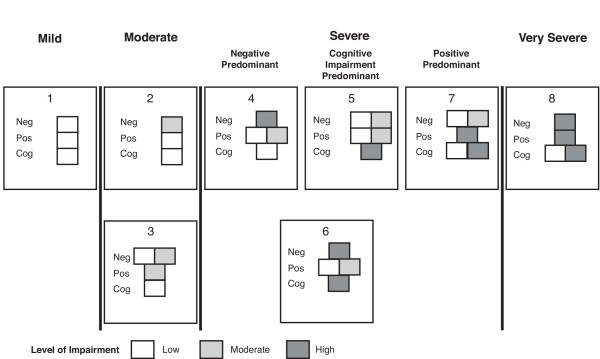
**Symptom description for the eight health states used in the PANSS-based mapping function**. PANSS indicates Positive and Negative Syndrome Scale; Neg, negative symptoms; Pos, positive symptoms; Cog, cognitive impairment; MOD, moderate symptom impairment. Adapted from Mohr PE, Cheng CM, Claxton K, et al. [16]. Reproduced with permission.

**Figure 2:**
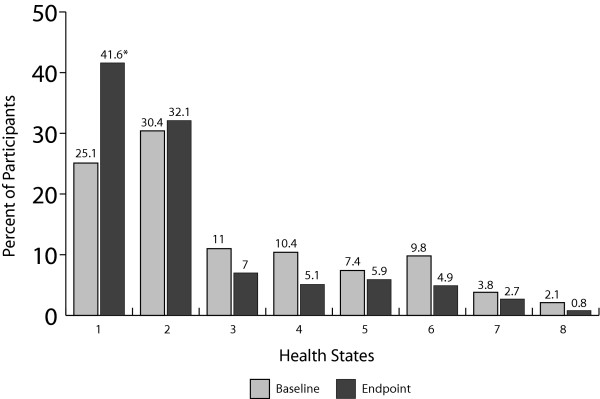
**Distribution of health states at baseline and at endpoint of the 1-year study**. Numbers of patients evaluated were 471 at baseline and 474 at endpoint. This figure illustrates both the floor effects of the measurement model as well as its descriptive validity: the percentage of patients in health state 2 shifted to a higher level of health in state 1 at the study endpoint. *P < 0.001 vs baseline, McNemar's test.

A graphic depiction of the eight health states used in the PANSS-based mapping function is provided in Figure 1. Each health state represents a set of symptoms ranging from mild to very severe, with patients having mild disease (health state 1) displaying low symptoms, and patients in the very severe disease state (health state 8) displaying high symptoms, with the exception of cognitive impairment, which could be either high or low. Two separate groups are considered to have moderate symptoms (health states 2, 3), while 4 health states (states 4–7) describe patients with severe symptoms [16]. The distribution of these health states at the beginning and endpoint of the trial are shown in Figure 2. Patients who completed treatment with long-acting risperidone experienced substantial symptomatic improvement over the 1-year study. Importantly, the percentage of patients in health state 1 (representing full remission of symptoms) increased significantly, from 25% to 42% over the course of the study (*P *< 0.001, McNemar's test). The impact of this shift was significant. Considering symptoms of schizophrenia alone, mean SG-weighted utilities increased significantly, from 0.729 at baseline to 0.775 at endpoint (*P *< 0.001, Wilcoxon signed rank test), with a net gain of 0.046. VAS-weighted ratings yielded similar results, with a gain in utility equaling 0.058 from baseline (0.538) to endpoint (0.596, *P *< 0.001, Wilcoxon signed rank test).

The incidence of common antipsychotic-associated adverse effects over the course of the study (parkinsonism, akathisia, dyskinesia, orthostatic hypotension, and weight gain) was assessed. Movement disorder side effects decreased over time, reflected in lower frequencies of moderate or severe parkinsonism (from 25.6% to 15.4%), akathisia (from 9.4% to 4.2%), and dyskinesia (from 13.6% to 9.5%) at endpoint. The occurrence of orthostatic hypotension overall was low; only 4 cases were reported during the study. Weight gain was the only adverse effect, with increased frequency over time. During the study, 51 patients gained ≥20 pounds, thus meeting the criteria for the utility tariff.

Adverse effects, as may be expected, impacted utility gains. Because adverse effects overall decreased with treatment over the course of the study, further gains in utility were realized (Table 2). Gains were 0.051 for SG-weighted comparisons and 0.064 for VAS-weighted comparisons after adjusting for adverse effects. Changes in utility from baseline to endpoint were statistically significant for both comparisons (*P *< 0.001, Wilcoxon signed rank test).

**Table 2:** Utility Gains Adjusted for Adverse Effects

	**VAS**	**SEM**	**SG**	**SEM**
Baseline	0.519	0.00725	0.712	0.00578
12 weeks	0.570*	0.00676	0.751^†^	0.00560
24 weeks	0.583*	0.00668	0.762^†^	0.00542
36 weeks	0.594*	0.00658	0.768^†^	0.00551
50 weeks	0.591*	0.00668	0.766^†^	0.00554
Endpoint	0.583*	0.00647	0.763^†^	0.00552

VAS indicates visual analog scale; SEM, standard error of the mean; SG, standard gamble.

*P < 0.001 versus baseline visual-analog-scale measurement (Wilcoxon signed rank test).

^†^*P *< 0.001 versus baseline standard-gamble measurement (Wilcoxon signed rank test).

**Figure 3:**
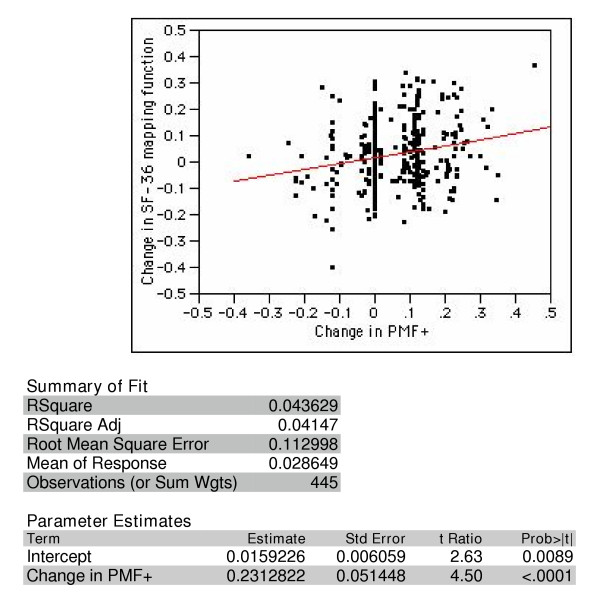
**Correlation between gains in utility, estimated using PANSS mapping function adjusted for averse effects (PMF+) and the SF-36 mapping function**.

Utility changes were estimated by a second method, which used the approach devised by Nichol and colleagues of mapping SF-36 domain scores to Health Utility Index (HUI) Mark II scores [8]. By this method, we found the average baseline utility to be 0.762. As with the disease-specific PANSS by adverse-effect method, utility attributable to long-acting risperidone treatment increased at endpoint but by a smaller degree, 0.0285 units (95% confidence interval: 0.039–0.017). The SF-36 mapping function was significantly but not strongly correlated with the PANSS by adverse-effect mapping function (r = 0.20 Pearson correlation coefficient; Figure 3).

To compare the responsiveness of both measures, we estimated the sample size that would be required for a randomized clinical trial to have 80% power to detect the changes in utility found in this observational study, at an alpha of 0.05. The standard deviation of the clinical mapping function was 0.127 at baseline and 0.125 at endpoint. The change in observed utility was 0.051, or about 0.4 standard deviations (a moderate effect, according to Cohen [24]). This translates to a requirement for about 156 total subjects to achieve the specified power. The change in utility seen with SF-36 function was smaller (0.0285), and the standard deviation was slightly larger (0.136 at baseline and 0.132 at endpoint). This translates to an effect size of 0.211, or about half the effect size of that seen with the clinical mapping function. A clinical trial designed to detect the observed change with the SF-36 mapping function would need 672 total subjects, or about four times the number that would be required if the study used the clinical mapping function. By way of comparison, change score for relevant PANSS items was 6.9 with a standard deviation 9.92. This translates to an effect size of .70. Only 19 subjects would be required to detect a positive change in the overall score.

## Discussion

Generation of utility weights for cost-effectiveness analysis is often a difficult task. This analysis applied a mapping function for the PANSS, with preference weights from a diverse sample of the US population, to a clinical observational study. Results demonstrate both the feasibility and the responsiveness of the function as a tool in cost effectiveness analysis. Estimates of gains in utility based on the disease-specific mapping function ranged from 0.046 to 0.064, depending on the scaling method and whether adverse effects of medication were included in the model. The effect was greater than that calculated using an SF-36-based mapping function, and the disease-based measure had greater precision and power to detect differences observed with treatment; however, its power was still not close to the change score for the PANSS items used in the mapping function.

These data confirm that utility calculations from disease specific and generic instruments may not be directly comparable. The relatively low correlation (r = 0.2) is probably due to the instruments covering different content areas. It could be argued that the optimal mapping system might incorporate both health status effects and disease effects in a utility model. To address the issue of avoidance of double counting of gains, one would need to apply methods to address the correlation that does exist between symptoms and their effects on health related quality of life. This might be done at the model formulation level through use of principal components analysis and cluster analysis to define states using both PANSS and SF-36 data. Methods described by Sugar and co-authors [25] might be suitable for this task.

Nonetheless, the estimates provided by the clinical function are better suited to use in a cost-effectiveness analysis than the ones derived from the SF-36 mapping function in this domain. The PANSS records an interviewer's perceptions of disease effects on the patient. The SF-36 is a self administered instrument. If an individual lacks the insight to appreciate health related quality of life impacts (lack of insight into disease effects is common in schizophrenia), then mapping functions based on self-report data might lack construct validity.

In mental illness disease effects and health related quality of life are highly convolved and it would be difficult to separate health related quality of life from disease experience. The clinical function was based on health state descriptions that included impacts of the disease on health related quality of life [17]. These descriptions were designed to be sufficiently comprehensive of health related quality of life to warrant direct usage in a cost-utility analysis. If descriptions had been limited to disease effects, further adjustments to utility estimates might be necessary prior to use in a cost-effectiveness analysis [26].

A few studies provide comparisons of utility gains with treatment in schizophrenia. Rosenheck and colleagues constructed a mapping function for schizophrenia with a quality-adjusted-life-year (QALY) like weight, based on subjectively defined "best" and "worst" possible health states [27]. They estimated that treatment of refractory patients with clozapine increased the quality-of-life measure by 0.049 units during a 1-year study. Pyne and coworkers estimated the utility gain with clinical improvement using the QWB scale and Brazier's mapping function for the SF-36 [28]. They found that "clinically significant" improvement in schizophrenia was associated with a 0.048 gain in utility using the QWB scale, and a 0.043 gain using the VAS-scaled version of Brazier's SF-36 mapping function.

This study had several limitations. First, the data were from an open-label study, which began with a 2-week oral risperidone run-in period. Estimates of gain in utility depended on the degree of symptom control that was achieved during this oral-dosing period; if symptoms were poorly controlled during this period, benefits of long-acting risperidone treatment for this population of clinically stable patients could have been overestimated. Second, this analysis included only patients who completed the trial. However, baseline demographics and disease characteristics of patients who completed the trial versus those who discontinued were not significantly different, with the exception of mean patient age (Table 1). While these design limitations limit the generalizability of findings of utility gains for treatment with long-acting risperidone, they do not impact assessments of the mapping function. Another important limitation of the clinical mapping function is the limited set of adverse effects of antipsychotic treatment accounted for in this model. While not all adverse effects were included in the mapping function, the features included have been described as the key benefits or liabilities of atypical agents versus conventional antipsychotics [29,30]. Thus, the most important and relevant medication side effects for contemporary pharmacoeconomic analyses have been included; however, the model may need to be expanded as new drugs are developed.

The mapping function applied in this study has technical advantages and disadvantages. The health states are based on a combination of clinical data and expert judgment. We believe that this is an optimal mix because it is a data-driven approach that compensates for under-representation of certain types of patients in clinical trials [16]. A second advantage is the software program used to elicit utilities. The software used multimedia video clips to describe the health effects of schizophrenia and adverse effects. This most likely improves the face validity of measurements because the health effects of schizophrenia can be difficult to comprehend to those without direct experience. A second advantage of the software program is its use of advanced methods for error correction in utility elicitations that have been proven to yield more accurate population estimates of utility values [31]. However, the computerized approach also brings limitations. Computer surveys are difficult to administer to "representative" samples. To limit data collection costs for the model, data were measured in members of an Internet survey panel [19]. Although participants were a diverse group in terms of geography, age, and ethnicity, the sample may not be representative of the US population because they were all Internet users (and members of a research panel) and because of drop-out due to technical issues with survey software.

## Conclusion

In summary, this paper describes the application of a new disease-specific utility mapping function, based on the PANSS and adverse events, to estimate gains in utility in a clinical study. This function is easy to apply and appears to have greater precision than a SF-36-based mapping function. One of the greatest advantages of the disease-specific mapping function is that it uses data generally available in clinical trials for schizophrenia (PANSS), and thus it could have wide applicability.

## Authors' contributions

LAL designed the study, developed the analysis plan, contributed to statistical analyses, and drafted the manuscript. MR participated in the design of the study, contributed to the analysis plan, and helped draft the manuscript. CE performed statistical analyses and helped draft the manuscript. All authors have read and approved the final manuscript.
